# Repository on maternal child health: Health portal to improve access to information on maternal child health in India

**DOI:** 10.1186/1471-2458-13-2

**Published:** 2013-01-02

**Authors:** Rajesh Khanna, N Karikalan, Anil Kumar Mishra, Anchal Agarwal, Madhulekha Bhattacharya, Jayanta K Das

**Affiliations:** 1 Saving Newborn Lives, Save the Children, New Delhi, India; 2 National Child Health Resource Centre (NCHRC), National Institute of Health and Family Welfare (NIHFW), Munirka, New Delhi 110063, India; 3 National AIDS Research Institute, Pune, India; 4 Combat Vehicles Research & Development Establishment, Ministry of Defense, Chennai, India; 5 NCHRC, NIHFW, New Delhi, India; 6 Department of Community Health Administration, NIHFW, New Delhi, India; 7 NIHFW, New Delhi, India

**Keywords:** Health information, Information management, Access, Health portal, Digital repository, Repository on maternal child health, India

## Abstract

**Background:**

Quality and essential health information is considered one of the most cost-effective interventions to improve health for a developing country. Healthcare portals have revolutionalized access to health information and knowledge using the Internet and related technologies, but their usage is far from satisfactory in India. This article describes a health portal developed in India aimed at providing one-stop access to efficiently search, organize and share maternal child health information relevant from public health perspective in the country.

**Methods:**

The portal ‘Repository on Maternal Child Health’ was developed using an open source content management system and standardized processes were followed for collection, selection, categorization and presentation of resource materials. Its usage is evaluated using key performance indicators obtained from Google Analytics, and quality assessed using a standardized checklist of knowledge management. The results are discussed in relation to improving quality and access to health information.

**Results:**

The portal was launched in July 2010 and provides free access to full-text of 900 resource materials categorized under specific topics and themes. During the subsequent 18 months, 52,798 visits were registered from 174 countries across the world, and more than three-fourth visits were from India alone. Nearly 44,000 unique visitors visited the website and spent an average time of 4 minutes 26 seconds. The overall bounce rate was 27.6%. An increase in the number of unique visitors was found to be significantly associated with an increase in the average time on site (p-value 0.01), increase in the web traffic through search engines (p-value 0.00), and decrease in the bounce rate (p-value 0.03). There was a high degree of agreement between the two experts regarding quality assessment carried out under the three domains of knowledge access, knowledge creation and knowledge transfer (Kappa statistic 0.72).

**Conclusions:**

Efficient management of health information is imperative for informed decision making, and digital repositories have now-a-days become the preferred source of information management. The growing popularity of the portal indicates the potential of such initiatives in improving access to quality and essential health information in India. There is a need to develop similar mechanisms for other health domains and interlink them to facilitate access to a variety of health information from a single platform.

## Background

Health information is important for planning, monitoring and improvement of services for the health of population. They are essential for policy-makers and programme planners to inform their decisions about what actions to take and what services to provide in order to improve the health of the populations they serve [1-3]. Equally important is information for health care recipients about the various preventive, promotive and curative aspects of illnesses, and the existing interventions and services available under the country’s health systems. Though significant effort has been made in providing health information to a growing number of people over the last decade, the progress has been patchy both geographically and across different health sectors [4].

Information and communication technologies (ICTs) are defined as tools that facilitate communication and the processing and transmission of information and the sharing of knowledge by electronic means. They have the potential to make a major contribution to improving access and quality of health services while containing costs [5]. The role of ICTs in health is usually focused on improving one of the following three domains:

i) the management of health information systems and access to that information

ii) communication about health, including improved information flows among health workers and the general public, better opportunities for health promotion and health communication, and improved feedback on the impact of health services and interventions

iii) the delivery of health care through better diagnosis, better training and sharing of knowledge among health workers, and supporting workers in primary health care, particularly rural health care [5]

One of the ICT tools that have revolutionalized access to health information and knowledge using the Internet and related technologies are Healthcare portals or E-health. They typically contain features that allow online health information seekers to be better informed and connected with other like-minded people. Their primary purpose is to facilitate improvements in quality and efficiency of healthcare by providing online educational information to the users in the form of discussion forums, useful articles, newsletters, interactive tools, and other useful health related resources [6].

As healthcare portals are increasingly accessed by the public, concerns have been raised about the ramifications of inadvertently publishing erroneous health information. The issue of quality of information becomes more important since large amount of information is available freely and easily (especially for those with connectivity). These concerns have created interest among the healthcare professionals to develop tools for assessing the accuracy of health information [7]. In the last decade, several rating systems have been developed to assess the accuracy of information in health portals; however many of these approaches have methodological limitations [8]. At the same time, people have expressed desire to build capacity of health workers so as to enable them distinguish unreliable from reliable sources of health information, and play an important role in educating patients to be critical users of the information they find on the Internet [4].

### Health information in India

India faces an enormous challenge in the area of maternal and child survival and contributes to the world’s greatest burden of maternal, newborn and child deaths. It also has the greatest number of undernourished children, and more than 50 million stunted children less than 5 years of age [9]. Under the mandate of Millennium Development Goals (MDG) 4 and 5 to reduce child mortality and maternal mortality, several initiatives had been undertaken by the government and non government organizations to improve the status of Maternal Child Health in India, including the National Rural Health Mission launched in 2005. These initiatives have generated an abundant resource of valuable information which however lies scattered or is not shared with different stakeholders.

Providing quality and essential health information is considered as one of the most cost effective intervention for a developing country like India [10]. A report says that 2/3 rd of the 50 million deaths could be saved just by application of knowledge [11]. However the availability of health information in India is inequitable and far from satisfactory. A review by Rabban et al. identified significant gaps in the availability of essential health information in public domain on the internet in India. The study found inequitable distribution and access to health information with public sector information readily available and negligible information available from the private sector which is a major source of healthcare in the country. Similarly national and state level health information was easily available but district-level information was limited [12].

Recently a national initiative has been undertaken to improve access to maternal child health information in India. Led by the National Child Health Resource Centre (NCHRC) at the National Institute of Health and Family Welfare (NIHFW) New Delhi, an online health portal known as the ‘Repository on Maternal Child Health’ (http://www.childhealthindiainfo.com) was developed which aims to provide one-stop access to efficiently search, organize and share maternal child health information relevant from public health perspective in India. This article briefly explains the development of this portal, analyzes its usage over an 18 month period, evaluates its quality using a standardized checklist of knowledge management, and discusses the results in relation to achieving improved access to health information.

## Methods

### Development of repository

NIHFW is an apex technical institute of the Ministry of Health and Family Welfare Government of India (MoHFW, GOI), for the promotion of public health and family welfare programmes in the country. The National Child Health Resource Centre was established at the institute in the Year 2008 to mainstream child health agenda in public health through collation, development, analysis and dissemination of relevant information to stakeholders at various levels, and create platform for discussion and information sharing on relevant issues at national level.

Though earlier studies had shown inadequate availability of health information in the public domain and lack of open access initiatives for usage of public health information in India [12,13], the studies were carried out before 2008 and there was a possibility of new initiatives being undertaken during the subsequent time period. So a detailed in-house assessment was carried out in October 2009 to identify the existing health information portals. After identifying them through web-surfing and literature search, the portals were evaluated with reference to five main criteria – types of information, accessibility & costs, presentation, target audience, and any special feature (Table 1).

**Table 1:** Detailed in-house assessment of health portals

**S. No**	**Name**	**Source/Host**	**Accessibility**			**Key areas of information**	**Target audience**	**Presentation**	**Remarks**
			**Free/Paid**	**Registration required**	**Full text available**				
1.	WHO-India Digital Repository	The Institutional Repository is an archive of WHO documents & resource materials from the WHO Country office India.	Free	No	Full text available and is divided into sections.	WHO resource materials on health	Professionals looking for WHO resource materials.	The information is divided into number of communities and sub-communities (or categories)	Limited information in every community.
						Main communities include Communicable diseases and disease surveillance, Family and Child Health, Health action in crisis, Health systems development, Immunization and Vaccinedevelopment, Non-communicable diseases and Mental health, Resources for staff members, Sustainable development			Materials limited to those developed by WHO only.
									No specific category for Child health.
2.	National Health Information Collaboration (NHIC)	NHIC is a collaborative model whereby the information is entered by authorized institutions and personnel from 18 organizations trained for the purpose.	Free	No	Full text available	Total of about 1800 entries (except Directories) out of which more than 1500 are scientific articles and thesis.	Research scientists, Healthcare professionals and Policy makers.	Since nformation is submitted through the authorized content providers only, limited resource materials or subject specific materials have been uploaded	First version and currently being updated.
						Classification based on Topics (all public health related) and types of information.			Child and adolescent health has 60 entries with 50 being scientific articles.
						Types include Scientific publications, Education & management, IT applications, Research projects & funding, Policies & practices, Stat data & Directory of resources.			
3.	Policy Reforms Option Database (HS-PROD)	Database is regulated by **PROD Management Group** made of health experts from the Govt and Non-Govt Sectors.	Free	Optional	No, only the summary of the case studies is available.	Health sector reforms – successful examples from the field and innovative ideas	Government of India Health and Family Welfare staff at central, state and district levels, health related NGOs, private sector providers, development partners (including donors) and academic institutions.	The information is provided using a standard format.	Includes case studies and innovative ideas on health sector reforms only.
		This group approve each entry before inclusion and meet quarterly to oversee progress.				Topics include Infrastructure, Logistics, Financial management, M&E, PPP, Management structures & systems, Social marketing, Health financing, Human resources, Urban health, Community participation, FRU, BCC			No separate topic on child health
4.	NGO Gateway	A website supported by NACO and UNAIDS.	Free	No	Yes	HIV/AIDS	Seeks to empower NGO sector through networking and capacity building by leveraging internet technologies.	It’s difficult to locate the documents for a new user.	Contains information related to HIV/AIDS only.
						Almost 1000 digital resources are present which have been categorized into Statistics/Data, Laws and Policy Documents, Information Resources, Tools, Funding Opportunities, and Declarations			

Some good websites were identified related to HIV-AIDS (NGO Gateway) and Health sector reforms (HS-PROD). There were other portals with sections on maternal child health information, but these were either not updated or contained limited information or were institutional portals with access limited to personnel working within these organizations. Thus our evaluation failed to identify a single website where maternal child health information was collated, managed and disseminated in an effective manner.

With this background information, the work on developing information portal started in December 2009 using the open source content management system Drupal [14]. A project plan with well defined outputs and timelines was prepared. In order to ensure quality of the portal, mechanisms and standardized processes were developed regarding the collection, selection/review, categorization and presentation of resource materials. Certain criteria were developed to ensure selection and dissemination of quality information and these include (1) Published material available in the public domain, (2) Source of publication known and trusted (Government, UN agencies, bilateral partners, academic and research institutes, reputed NGOs, etc.), (3) Peer-reviewed material, (4) No personal opinion or commentary, and (5) Administrative rights to upload material restricted to selected team members. These criteria acted as a strong filter to prevent sharing of inaccurate data. All the mechanisms including the criteria were shared with technical experts from the Government and Non-governmental organizations and appropriate changes made based on their feedback.

Health information was collected from various resources including internet searching and contacting 900 organizations through emails and letters. This work took longer than expected due to non-response from majority of organizations and reluctance to share their resource materials. Efforts were made to personally contact these institutions/libraries through visits and telephone calls. The resources were scrutinized for eligibility using pre-defined criteria; those selected were categorized under specific topics and themes, and presented in an abstract form with key words and a thumbnail picture of the cover page. Emphasis was laid on providing access to updated information from Year 2000 onwards, and on providing access to full text documents for all the resource materials.

The major topics included Maternal Health, Newborn Child Health, Nutrition, Immunization, Diseases and General Information. Each topic was further sub-categorized to include variety of resources for the different groups of target audiences – *Policies, Programmes, Guidelines* for those involved in planning and policy making, *Technical publications* (reports, case studies, advocacy, etc.) for those in programme development and management, *Training materials* for capacity building of health workers, *Statistical information* for those involved in monitoring evaluation and research, and *Scientific articles* for academicians, researchers and policy analysts. The website was launched in July 2010.

### Analysis of usage

The Repository usage was analyzed using Key performance indicators obtained from the Google Analytics for the time period July 2010 to December 2011. Google Analytics is a free service offered by Google that generates detailed statistics about the site usage and provides some information about how the site can be improved further [15]. The performance indicators evaluated are the number of visits, absolute number of unique visitors, average pageviews per visit, bounce rate, average time spent on site, and the various traffic sources. While the number of visits and page views are general indicators of the traffic load, unique visitors reflect the actual number of unduplicated (counted only once) or ‘first time’ visitors to the website over a specified time period. Bounce rate is the percentage of single-page visits or visits in which the person left the website from the entrance/landing page. It is one of the key indicators reflecting the quality of website with a lower figure indicating better quality. A rate of less than 40% is considered ‘good’, while rate less than 20% is considered ‘excellent’ for any website [15]. Average time spent on the site also reflects the quality, however it is often misleading because the visitors leave their browser window open even when they are not using it. The traffic sources provide an overview of the different kinds of sources that direct traffic to the website [15].

We first calculated the indicators for the entire 18 month duration. This was followed by analysis of the monthly records to identify trends of performance indicators. The number of absolute unique visitors was taken as an independent variable and its association with other indicators (average pageview per visit, bounce rate, average time spent on site) evaluated using linear regression analysis. Statistical package SPSS-19 was used for the analysis and P-value < 0.05 accepted as level for statistical significance.

### Quality assessment

Quality assessment was carried out independently by two external Knowledge Management experts using a standardized checklist tool [6]. The two experts were identified from the list of technical experts available with the institute. The two were unknown to each other and blinded to each other’s response. To measure agreement between the two experts, Cohen’s kappa test was used.

The standardized checklist used for the assessment helps to evaluate the website in three key domains of knowledge access, knowledge creation and knowledge transfer. There are 52 features or questions grouped under dimensions and sub-dimensions for assessing the three domains. *Knowledge Access Mechanism* refers to the mechanisms through which visitor’s access the portal and its information – searching, browsing, personalization, accessibility options and multi-lingual support. This mechanism highlights the importance of tools that are able to integrate capabilities for searching information with greater precision and tools that are able to personalize the content and presentation of the interface. The second mechanism of *Knowledge Creation* refers to the process of capturing visitor’s information such as demographics, preferences and behaviors and creating new knowledge that will benefit portal providers and the users. The mechanism emphasizes techniques and processes that are able to capture visitor’s information and create value from it. The third mechanism, *Knowledge Transfer Mechanism,* refers to the mechanisms that allow the portal providers to foster user-to-user and provider-to-user sharing of knowledge. Specifically, this mechanism highlights tools that enable user to share their knowledge with fellow users as well as tools to interact directly or indirectly with experts, advisors, and customer representatives [6].

## Results

### Usage and trends

From July 2010 to December 2011, there were a total of 52,798 visits to the website from 174 countries across the world. More than three-fourth of the visits (77%) came from India alone, followed by the United States of America (5%). In India, the visits came from 85 cities. During the 18 month period, a total of 292,652 pages were viewed giving an average of 5.54 pages viewed per single visit. Nearly 44,000 unique visitors visited the website and spent an average time of 4 minutes 26 seconds. The overall bounce rate was 27.6%. Nearly two-thirds of the traffic came from the Search engines, one-fifth from the referring sites, and the remaining was Direct traffic.

**Figure 1:**
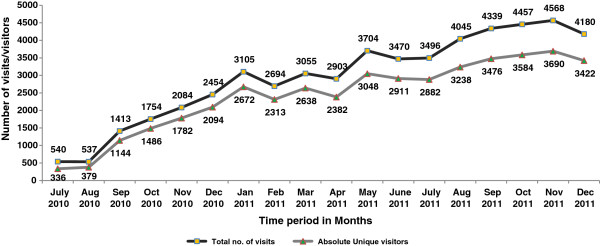
**Displays the monthly trend of number of visits and number of absolute unique visitors during the 18 month period.** It was observed that there was a consistent increase in the number of visits and the number of unique visitors during this time. While the number of visits and unique visitors during July 2010 were 540 and 336 respectively (first month after launch), the figures rose to 2454 and 2100 after 6 months, to 3500 and 2900 at 1 year, and to 4200 and 3400 respectively at 18 months.

Table 2 gives the monthly figures of key performance indicators seen during the assessment period. A consistent increase in the number of visits and the number of unique visitors was observed during this time (Figure 1). While the number of visits and unique visitors during July 2010 were 540 and 336 respectively (first month after launch), the figures rose to 2454 and 2100 after 6 months, to 3500 and 2900 at 1 year, and to 4200 and 3400 respectively at 18 months, that is, in December 2011.

**Table 2:** Monthly distribution of key performance indicators of repository (July 2010 – December 2011)

							**Traffic sources**		
**Month**	**Total no. of visits**	**No. of Abs. unique visitors**	**Bounce rate (%)**	**Total no. of pageviews**	**Page view per visit**	**Avg. time on site (min.sec)**	**Search engine (%)**	**Referring site (%)**	**Direct traffic (%)**
**Jul-10**	540	336	52.96	1640	3.04	4.38	19.63	51.48	28.89
**Aug-10**	537	379	24.95	5543	10.32	11.14	28.12	44.88	27.00
**Sep-10**	1413	1144	21.94	8217	5.82	4.40	34.89	37.08	27.95
**Oct-10**	1754	1486	37.29	9184	5.24	5.45	59.18	23.60	17.22
**Nov-10**	2084	1782	44.00	9931	4.77	6.23	60.60	18.28	21.11
**Dec-10**	2454	2094	44.25	11540	4.70	3.57	69.15	16.87	13.98
**Jan-11**	3105	2672	46.28	12651	4.07	3.47	69.86	14.98	15.17
**Feb-11**	2694	2313	43.10	11047	4.10	3.39	67.45	16.26	16.30
**Mar-11**	3055	2638	45.17	11605	3.80	4.03	62.09	16.82	21.08
**Apr-11**	2903	2382	40.61	11428	3.94	4.18	52.26	27.49	20.25
**May-11**	3704	3048	37.53	15677	4.23	4.44	51.00	27.89	21.11
**Jun-11**	3470	2911	38.70	16775	4.83	5.05	52.28	26.83	20.89
**Jul-11**	3496	2882	40.53	14951	4.28	4.38	56.09	22.77	21.14
**Aug-11**	4045	3238	18.02	25757	6.37	3.55	71.52	16.84	11.64
**Sep-11**	4339	3476	9.33	33800	7.79	4.28	73.75	17.67	8.58
**Oct-11**	4457	3584	6.87	33094	7.43	4.35	73.27	17.59	9.13
**Nov-11**	4568	3690	4.93	30572	6.69	3.33	79.09	12.94	7.97
**Dec-11**	4180	3422	5.69	29240	7.00	3.57	82.22	11.65	6.12
**Total for 18 months (Jul 2010 – Dec 2011)**	52798	43477	27.65	292,652	5.54	4.26	64.81	19.96	15.23

**Figure 2:**
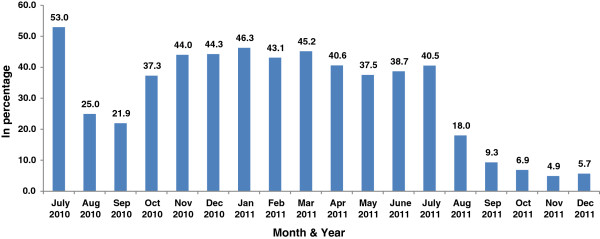
**Shows the monthly distribution of the bounce rate.** There was an initial decrease in the bounce rate during the early part of the 18 month period followed by an increase which however again decreased during the latter half of the reporting period. The relevant monthly figures during the last 4 months were all less than 10%.

An increasing trend was also observed in the average number of pageviews per visit especially during the latter half of the assessment period. For the bounce rate, an initial decrease was followed by increase in the rate which however again decreased during the latter half of the reporting period. The relevant figures during the last 4 months were less than 10% (Figure 2). The average time spent by a visitor usually ranged between 3 to 5 minutes.

The source of web traffic was found to change dramatically during the 18 months. At the beginning, most of the traffic came from the referring sites of the organization. Gradually search engines (Google) became more important source of web traffic and during the last 4 months, nearly 75-80% of the traffic came from it.

Table 3 shows the association between number of unique visitors and the other indicators. There was a statistically significant association between increase in the number of unique visitors and increase in the average time on site (p-value: 0.01) and web traffic through search engines (p-value: 0.00). The increase in number of unique visitors was also significantly associated with a decrease in the bounce rate (p-value: 0.03).

**Table 3:** Association of number of absolute unique visitors with other key performance indicators

**Performance indicators**	**Absolute unique visitors**				
	**α**	**B**	**R**	**t**	**p-value**
Bounce rate	50.132	−0.008	0.505	−2.337	0.033
Page view per visit	5.200	0.000	0.063	0.252	0.804
Avg. time on site	7.071	−0.001	0.585	−2.889	0.011
Search engine	25.192	0.014	0.840	6.196	0.000

### Quality assessment

For each checklist item, the response could be ‘Yes’ or ‘No’ based on whether the relevant feature was supported by the website. Out of the 52 items, one of the experts had not responded for 3 items and hence these items were not counted while evaluating agreement (Table 4). The value for Cohen’s Kappa statistic was 0.72 suggesting a high degree of agreement between the two experts.

**Table 4:** 2x2 Table displaying the agreement between two external reviewers

		**External 2**		**Total**
		**Yes**	**No**	
External 1	Yes	22	6	28
	No	1	20	21
Total		23	26	49

Value of Cohen’s Kappa Statistics: 0.72.

For the Knowledge Access Mechanism, searching, browsing, accessibility options and multi-lingual support were found to be adequately covered. The repository appeared as the first choice under Google when exact name was used for searching, and was displayed on the first page when searched with other key terms like maternal child health resources and information on maternal child health. Facility for free text search was available with options for advanced search, recommended searches and sorting of articles. Glossary, sitemap and an index were provided to facilitate browsing. Information was presented using images, video and audio files, and multi-lingual support was given. However there were inadequate efforts to enable personalization of information for individual users and organizations, and to ensure accessibility of portal for people with disabilities.

The items covered under the mechanism of Knowledge Creation, that is capturing user’s information (demographics, preferences, and behaviors) and creating new knowledge to benefit the host organization and end-users, were found to be deficient except for a provision to capture feedback from the end users.

Under the third mechanism of Knowledge Transfer, the portal was observed to enable sharing of information between the users through the facility of emailing web links/pages to a friend, giving tips on search via frequently asked questions, and providing live demonstration on how to use the website. New information was found to be displayed as an important alert. The portal had its own catalogue of information which was linked to other websites for sharing. However the other possible mechanisms for knowledge transfer (ask an expert, discussion forums, blogs, wiki support, instant messaging, online group service, online chat) were not available.

## Discussion

This study describes the development and utilization of a national portal of information on maternal child health developed in India using open source technology. There was a growing popularity of the portal as seen by a consistent increase in the number of visits and absolute unique visitors during the 18 month period. While the average time spent on the portal and the average page views remained consistent during the period, there was decrease in the bounce rate. Noteworthy was the bounce rate of less than 10% observed during the last quarter, which suggests a large number of users accessing the website for information and not leaving it from the home/landing page. Certain promotional activities undertaken during the last quarter (printing and distribution of a brochure, informing about new materials through existing discussion forums) could be the reason for this dramatic change. A significant association was also observed between increase in the number of unique visitors and decrease in the bounce rate indicating an increasing usefulness of the portal. The most common mode of accessing the website was the Google search engine.

The World Health Organization has recognized equitable and universal access to health-care information as an important part of worldwide strategies to reduce global disparities in health and to achieve the health related MDGs [16]. A key paper produced as part of a global review on access to health information concluded that ‘Universal access to information for health professionals is a prerequisite for meeting the MDGs and achieving Health for All [4]. Our results show gradual increase in access to health information especially within India. While the portal was accessed across 174 countries worldwide, more than three-fourth of the total visits came from India alone. Within the country, the website was accessed across 85 cities and there was a consistent increase in the number of visits and unique visitors. However it was difficult to evaluate the accessibility of the portal at the district level and by the community as user’s information was not being captured.

**Figure 3:**
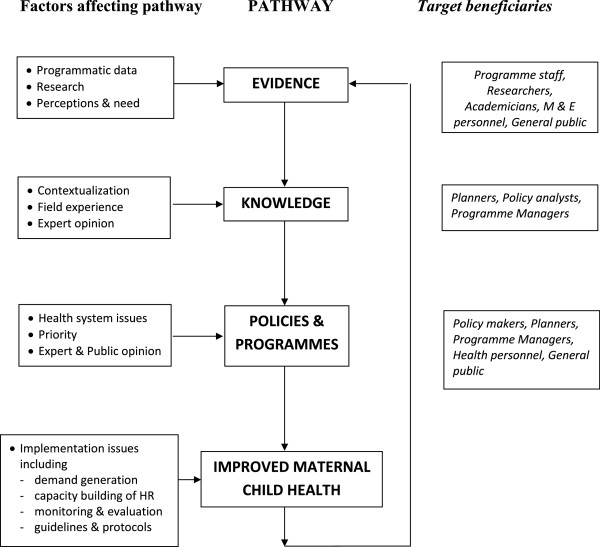
Theory of change for repository role in improving maternal child health.

Apart from increasing usage and accessibility of health information, the portal can play a key role in facilitating the process of evidence-informed policy making which would contribute to improved maternal child health in the country. The proposed ‘theory of change’ pathway is illustrated in Figure 3. It can be seen that the health portal can make a significant contribution to each of the three key steps of evidence generation, translation of evidence to knowledge, and development and dissemination of evidence-informed policies and programmes.

Various successful initiatives undertaken in the past like the BIREME (Brazil), EMRO, NML-ERMED (India), and HINARI (WHO) have taught important lessons in promoting equitable access to health information. This includes providing information according to the need of the end user (‘pull’ out the required information rather than ‘push’ in irrelevant information) and through a variety of resources (evidence-based handbooks, guidelines, training manuals, etc. rather than limiting it to scientific journals only), building local capacity for sustainable development, ensuring free access and usage of ‘open access’ technology, and trying to sustain interest of the funding agencies (through specific measurable outputs) [4].

During the development of the Repository, an effort was made to incorporate the above mentioned lessons. A wide variety of resources were made available depending upon the information needs of different health personnel as evaluated in an earlier study from the Indian state of Uttar Pradesh [17]. These included evidence-based policies, technical reports and best practices required for policy planning at national level, state/district level data required for programme planning and guidelines for programme implementation, and behavioral communication change materials required for informing and motivating the community. In addition, number of other resources including training modules & manuals, advocacy materials, scientific articles on primary/secondary research, audio and video health promotion materials, were also included. The complete process of portal development, customization and presentation was also carried out within the organization in order to build the capacity of the team and ensure sustainability of the project.

The team had some additional learning. There was lack of acceptance among the stakeholders including healthcare professionals and organizations leading to their reluctance in sharing resources. However this changed after one year of development and was facilitated by the Government’s (MoHFW) support given during events and meetings. Promotional activities helped to increase the portal usage and had to be carried out regularly. Maintaining the quality of the portal including uploading of new resource materials was a big challenge and required constant inputs from the team and external Knowledge Management experts.

Open access is an innovative mode of scholarly communication of this century which assures universal access to information and knowledge at total free of cost, provided the information seeker have an internet facility [18]. It is the third generation of publication mode next to text book and E-Publication. According to the National Knowledge Commission, it one of the most effective ways of reducing the information gap prevailing in India [19]. Various studies have shown the positive impact of open access initiatives in improving public health status of population. While a WHO paper shows the positive impact of an open access article in the prevention of HIV through male circumcision [20], another study suggests the success of an Alzheimer’s disease research through open knowledge sharing which was successfully replicated in another research about Parkinson disease in network [21].

It has been said that India has been unable to explore the benefits of open access movement in the public health sector. According of the Directory of Open Access Repositories (DOAR), out of the 42 repositories in India, only one reflects about health and medicine and others are for science and interdisciplinary fields [13]. This could be due to lack of leadership, lack of acceptance for the open access technology or reluctance of stakeholders to share resources with each other. The Repository on Maternal Child Health was developed using an open source content management system Drupal. All the content uploaded on the portal are accessible without any registration and available free of cost to all end users. The success of the portal has also motivated stakeholders who were initially reluctant to share information, to send in their resource materials and facilitate sharing of information at the national level. During the last 4 months, 112 new resource materials were uploaded and 57 of these were shared by the stakeholders including 12 from the MoHFW, GOI.

There was a high level of agreement between the experts regarding the quality of the portal. While many features for Knowledge Access and Knowledge Transfer were observed to be present in the portal, features capturing visitor’s information such as demographics, preferences and behaviors were not included. This was done purposely to make the whole process simple and time-saving, and allow more freedom and unrestricted access to information. Other features for personalization, customization and registration (to capture user’s information) were also not included due to the same reasons. Multi-lingual feature and photograph of the cover page were added to facilitate access for the above mentioned target groups. In order to avoid duplication of efforts, no attempt was made to start a discussion forum as a vibrant discussion platform already exists in India (http://www.solutionexchange-un.net.in/).

In the future, we are looking into ways to improve the content presentation of the portal using interactive technologies, and translate the resources into regional languages so to improve its usage and accessibility in different parts of the country including rural areas.

## Conclusion

Our results show the growing popularity of the Repository on Maternal Child Health in India, an E-portal for information management developed using open source content management system. The portal contains a variety of resources for a wide range of stakeholders, and was successful in promoting access to health information. All the content is available free of cost. It was found to include many features to aid access and transfer of information between the users.

Efficient management of health information is imperative for informed decision making and for attaining effective programmatic outcomes. Digital repositories have now-a-days become the preferred source of information management. The success of the Repository on Maternal Child Health indicates the potential of such mechanisms to improve availability of health information related to other health domains also like nutrition, family welfare and adolescent health. Efforts should be taken to interlink these initiatives so as to facilitate easy access of all types of health information from a single platform.

## Availability and requirements

· **Project name:** Repository on Maternal Child Health

· **Project home page:**http://www.childhealthindiainfo.com/

· **Operating system(s):** Windows

· **Open source CMS:** Drupal

· **License:** Not required (open source software)

· **Any restrictions to use by non-academics:** None

## Competing interests

The authors declare that they have no competing interests

## Authors’ contributions

AKM & RK were involved in developing and customizing the health portal; AKM, NK & RK conceived the idea of a manuscript and developed the initial plan; AKM, NK & AA participated in data collection and analysis; NK, AA and RK helped in preparing the draft manuscript; RK, MB & JKD were involved in revising the draft manuscript and preparing the final article. All authors read and approved the final manuscript.

## Authors' information

Both AKM & NK had previously worked with NCHRC. AKM was the Library Information Officer and NK had worked as an Intern while doing his Masters in Public Health from.

## Pre-publication history

The pre-publication history for this paper can be accessed here:

http://www.biomedcentral.com/1471-2458/13/2/prepub
